# MG53 in Early Skeletal Muscle Stem Cell Activation: Implications for Aged Muscle Regeneration

**DOI:** 10.3390/cells15050463

**Published:** 2026-03-05

**Authors:** Yanping Xu, Jethro Wang Zih-Shuo, Zhentao Zhang, Peng Chen, Usman Alizai, Keerthika Sathish, Sakai Lilian, Zhiyu Yan, Bryan A. Whitson, Timothy M. Pawlik, Hua Zhu

**Affiliations:** Department of Surgery, The Ohio State University Wexner Medical Center, Columbus, OH 43210, USA; yanping.xu@osumc.edu (Y.X.); zishuo.wang@osumc.edu (J.W.Z.-S.); zhentao.zhang@osumc.edu (Z.Z.); peng.chen@osumc.edu (P.C.); usman.alizai@osumc.edu (U.A.); sathish.4@buckeyemail.osu.edu (K.S.); lilian.sakai@osumc.edu (S.L.); zhiyu.yan@osumc.edu (Z.Y.); bryan.whitson@osumc.edu (B.A.W.); tim.pawlik@osumc.edu (T.M.P.)

**Keywords:** skeletal muscle regeneration, satellite cell activation, aging, activation checkpoint, stress, MG53/*TRIM72*

## Abstract

**Highlights:**

**What are the main findings?**

- Early MuSC activation is discussed as a stress-sensitive transitional phase in skeletal muscle regeneration.
- Aging is associated with selective instability of this activation window, while downstream myogenic programs remain comparatively preserved.

**What are the implications of the main finding?**

- Age-related regenerative decline can be interpreted in part as reduced activation fidelity rather than uniform loss of myogenic potential.
- Stabilizing early activation dynamics is considered as a possible conceptual direction for future investigation.

**Abstract:**

Skeletal muscle regeneration declines with age despite the persistence of satellite cells (muscle stem cells, MuSCs), suggesting that regenerative impairment reflects functional dysregulation rather than MuSC depletion. Increasing evidence identifies early MuSC activation during the immediate post-injury period as a stress-sensitive, rate-limiting transition that is particularly vulnerable in aged muscle. Aged MuSCs exhibit elevated stress responses and reduced membrane remodeling capacity, accompanied by weakened activation-associated transcriptional induction. In contrast, proliferative and differentiation programs remain largely intact once activation is successfully initiated. These findings underscore that impaired coordination during early activation contributes to long-term regenerative decline in aging. Within this framework, MG53 (tripartite motif–containing protein 72, *TRIM72*), a muscle-enriched TRIM family E3 ubiquitin ligase originally identified as a mediator of sarcolemmal membrane repair, may also function as a stress-responsive regulator that stabilizes the early activation environment. Rather than directly determining cell fate, MG53 is proposed to facilitate activation by mitigating stress-associated membrane disruption and maintaining programmatic coordination under age-related physiological constraints. Most mechanistic evidence derives from rodent models, and direct validation in human aging muscle remains limited. These observations suggest that targeting early activation, rather than simply increasing proliferation, may better preserve regenerative capacity in aging skeletal muscle.

## 1. Introduction

Skeletal muscle regeneration depends on the timely activation and coordinated fate progression of satellite cells (muscle stem cells, MuSCs), which reside beneath the basal lamina and maintain tissue integrity across repeated bouts of injury and physiological stress [1,2]. In aged muscle, regenerative decline is a well-established hallmark; accumulating evidence indicates that this impairment cannot be explained solely by a quantitative loss of MuSCs [3,4]. Instead, multiple studies support a qualitative shift: MuSCs remain present but show diminished efficacy in executing the early steps required for productive regeneration [5,6,7,8].

MuSC activation represents a critical transitional process in which quiescent MuSCs break homeostasis and acquire regenerative competence [2]. This transition precedes cell-cycle entry and occurs upstream of canonical myogenic transcriptional programs [9]. Increasing evidence suggests that early activation constitutes a vulnerable checkpoint that integrates intrinsic cellular state with extrinsic niche-derived signals [3,10]. During this phase, MuSCs undergo extensive cytoskeletal remodeling, changes in adhesion and polarity, metabolic reprogramming, and induction of immediate early gene networks [10]. Failure to execute these events with appropriate timing and coordination can delay regeneration, bias fate trajectories, or compromise long-term MuSC function [5,6].

Aging profoundly alters the muscle microenvironment, introducing chronic oxidative stress, low-grade inflammation, and mechanical instability [11]. These perturbations place a disproportionate burden on early activation, increasing the frequency of delayed or abortive regenerative responses and accelerating MuSC attrition across repeated regenerative cycles [5,6,8]. Consequently, preserving the fidelity of early MuSC activation—rather than simply enhancing downstream proliferation or differentiation—has emerged as a central issue in understanding age-associated regenerative decline [3,12].

MG53 (tripartite motif–containing protein 72, *TRIM72*) is a striated muscle-enriched protein classically defined by its role in membrane repair in mature myofibers. In response to oxidative stress, MG53 undergoes redox-dependent oligomerization, enabling rapid recruitment of vesicular repair machinery to sites of membrane disruption [13,14,15]. In addition to its canonical membrane repair function, MG53 has been reported to function as a circulating myokine. Studies led by Dr. Jianjie Ma identified its interaction with tissue plasminogen activator (tPA) and described systemic roles in membrane protection and tissue repair [16,17]. Beyond skeletal muscle, MG53 has been implicated in protective responses across multiple organs—including lung [18,19], kidney [20,21], liver [22,23], heart [24,25], eye [26,27,28], and brain [17,29,30]—where it modulates inflammatory signaling and preserves membrane stability. Notably, Bian et al. reported enhanced MuSC proliferation in MG53–tPA-treated rodent models [16].

However, direct experimental evidence defining the role of MG53 in the early activation of aged MuSCs remains limited. Current data primarily support its functions in membrane stabilization, oxidative stress mitigation, and inflammatory modulation [31]. Whether these stress-buffering properties directly influence the early activation transition in aging muscle has not yet been formally tested. In this review, we suggest that MG53 may contribute to the regulation of early MuSC activation under conditions of elevated cellular stress in aged muscle. Clarifying this potential role represents an important direction for future mechanistic investigation.

## 2. The Aged MuSC Niche and Regulation of Early Activation

### 2.1. Classical MuSC States Revisited in Aging

**Figure 1:**
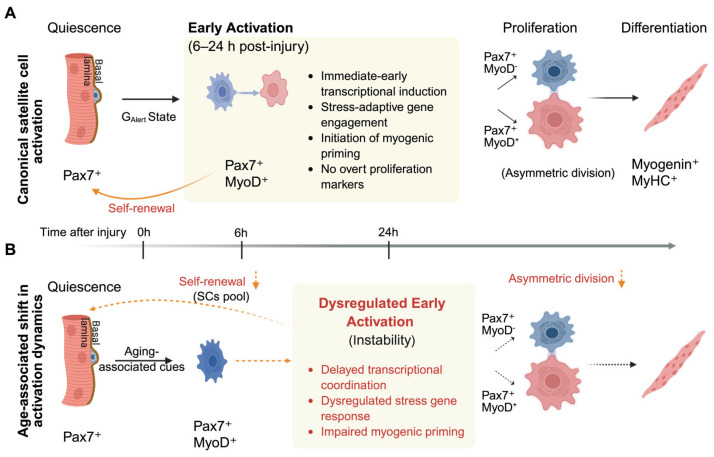
Early activation as a stress-sensitive checkpoint in young and aged MuSC regeneration. (**A**) Under young or homeostatic conditions, MuSCs reside in a quiescent Pax7^+^ state beneath the basal lamina. Following acute injury (0 h), MuSCs enter an early activation phase (6–24 h post-injury), characterized by immediate-early transcriptional induction, stress-adaptive gene engagement, initiation of myogenic priming, and absence of overt proliferation markers. (**B**) In aged muscle, MuSC numbers are relatively preserved; however, the early activation window becomes prolonged and transcriptionally dysregulated. Aging-associated stress disrupts coordinated gene induction and impairs myogenic priming, delaying efficient progression toward proliferation. This instability within early activation alters division dynamics (asymmetric division), reduces effective self-renewal, and compromises regenerative fidelity. Note: Blue-colored cells represent the quiescent or early activated state; red-colored cells indicate cells undergoing myogenic priming or proliferation. Abbreviations: MuSC, muscle stem cell; Pax7, paired box 7; MyoD, myogenic differentiation 1; MyHC, myosin heavy chain; G_Alert_, alerted state of quiescence. Created with BioRender.com (2026 version); https://BioRender.com/tuieb7q (accessed on 11 January 2026).

MuSCs maintain skeletal muscle integrity by residing quiescent under homeostatic conditions and rapidly activating in response to injury or stress. In murine injury models, canonical regeneration proceeds from quiescence through activation, proliferation, differentiation, and self-renewal, restoring myofiber structure while preserving the MuSC pool [11,32] (Figure 1A). However, this linear view underestimates the importance of the earliest regenerative transition. Exit from quiescence represents a discrete, tightly regulated activation phase that precedes cell-cycle entry and is not simply a passive precursor to proliferation [1,2]. Accumulating evidence supports a preparatory activation state in which MuSC integrate systemic, niche-derived, and intrinsic cues to establish regenerative competence [9,11,33]. Rodgers et al. showed that systemic stimuli in a mouse acute injury model can drive quiescent MuSCs into a metabolically primed G_Alert_ state via mechanistic target of rapamycin complex 1 (mTORC1) activation, enhancing regenerative readiness without inducing immediate proliferation [9]. Consistent with this view, in situ fixation studies revealed that early activation involves transcriptional reprogramming and cytoskeletal remodeling prior to overt cell-cycle progression [10]. Importantly, early activation is tightly coupled to division symmetry and long-term MuSC fate. Seminal work established asymmetric division as a core mechanism supporting MuSC self-renewal and regenerative sustainability [34]. Subsequent in vivo studies further showed that even modest shifts in the balance between asymmetric and symmetric divisions are sufficient to impair muscle repair [35]. These findings indicate that defects arising upstream of cell-cycle entry can compromise regenerative output, even when downstream proliferative or differentiation programs remain largely intact. Aging does not eliminate the regenerative sequence but alters how it is executed. In aged mouse muscle, MuSC numbers are largely maintained [4,36], yet exit from quiescence becomes slower and less coordinated. Age-related niche perturbations destabilize quiescence maintenance [37], while intrinsic changes bias MuSCs toward irreversible quiescence or senescence [8]. These alterations disrupt the timing and reliability of early activation, leading to impaired self-renewal and reduced regenerative efficiency. Thus, age-associated regenerative decline is more accurately viewed as a failure of activation fidelity rather than a loss of myogenic potential (Figure 1B).

### 2.2. Defining Early Activation as a Discrete Transitional State

Early MuSC activation, as defined in this review, denotes the immediate post-quiescent phase that precedes overt cell-cycle entry [1,10]. This interval is molecularly distinct from both quiescence and proliferation and is characterized by rapid transcriptional reprogramming, cytoskeletal remodeling, and induction of stress-adaptive pathways.

Operationally, quiescent MuSCs express canonical MuSC markers including *Pax7*, *Notch3*, *Spry1*, *Foxo3*, and *Calcr* [38,39,40,41]. Upon injury, early activation is marked by induction of immediate-early transcription factors (*Fos*, *Jun*, *Egr1*, *Atf3*) [10,42,43,44], stress-responsive genes (*Mt1/2*, *Hmox1*, *Socs3*) [43,45,46], and early myogenic regulators (*Myf5*, *Myod1*) [11,32], while remaining largely negative for proliferation-associated markers such as *Mki67*, *Ccnb1*, and *Pcna* [9,10,44,47,48]. Full cyclin upregulation and mitotic gene expression define the subsequent proliferative phase (see Table 1).

This definition distinguishes early activation from the G_Alert_ state described by Rodgers et al., which represents an mTORC1-dependent priming of quiescent cells without complete transcriptional remodeling or cell-cycle engagement [9]. Whereas G_Alert_ reflects a heightened quiescent readiness, early activation entails active reprogramming and initiation of regenerative commitment following injury.

In murine injury models, early activation predominantly occurs within the first 6–24 h after acute damage, prior to robust S-phase entry and clonal expansion [10]. During this window, chromatin accessibility and metabolic rewiring are initiated, but DNA replication and sustained cyclin induction have not yet occurred [42,44,49,50]. Thus, early activation represents a preparatory yet decisive checkpoint that determines subsequent regenerative trajectory [7,8].

**Table 1:** Operational gene sets defining stage-specific states of MuSCs during regeneration.

Regenerative Stage	Functional Definition	Representative Markers	Key References
Quiescence	Stem cell maintenance, niche anchorage, stress resistance	*Pax7*, *Notch3*, *Calcr*, *Foxo3*, *Spry1*	[38,39,40,41]
Early activation	Immediate-early transcription, stress adaptation, early myogenic priming	*Fos*, *Jun*, *Egr1*, *Atf3*, *Myf5*, *Myod1*	[10,11,32,42,43,44,45,46]
Proliferation	Cyclin induction, S-phase entry, mitotic progression	*Mki67*, *Ccnd1*, *Ccnb1*, *Pcna*	[9,10,44,47,48]
Differentiation	Terminal myogenic commitment and contractile program	*Myog*, *Myh3*, *Myh8*, *Mef2c*	[32,51]

The complete gene lists and literature mapping are provided in Supplementary Table S1.

### 2.3. Early Activation as a Stress-Sensitive Transitional Checkpoint in Aging

As discussed above, this interval serves as the gateway between quiescence and proliferation, its successful execution depends on tightly coordinated structural and transcriptional adjustments. Even modest disruptions in cellular homeostasis can compromise progression toward productive proliferation [42,44].

In vivo lineage-tracking and niche-manipulation studies show that activation trajectories vary across individual MuSCs and are shaped by local constraints [35]. Functional evidence further indicates that early activation can stall or fail to progress into productive proliferation when adaptive capacity is insufficient [9,10]. Importantly, such failure does not reflect loss of myogenic identity, but rather impaired coordination of activation dynamics.

Aging in murine models selectively amplifies this vulnerability. Age-associated alterations in the MuSC niche destabilize the quiescence-to-activation transition and increase the stress burden encountered during early activation [37], while intrinsic aging-related changes bias MuSCs toward irreversible quiescence or senescence, narrowing the margin for successful activation [8]. As a result, aged MuSCs are more likely to enter a prolonged and poorly synchronized activation state, reducing coordination across the population and impairing subsequent cell-cycle entry and self-renewal [33]. Viewing early activation as a regulated checkpoint therefore helps explain why aging preferentially compromises regenerative competence by degrading activation fidelity rather than eliminating the MuSC pool.

Therefore, accumulating evidence indicates that early activation represents a tightly regulated transitional phase shaped by the integration of intrinsic transcriptional induction, stress-adaptive signaling pathways, and niche-derived mechanical and inflammatory cues. Rather than constituting a passive prelude to proliferation, this interval involves coordinated chromatin remodeling, metabolic adjustment, and cytoskeletal reorganization that collectively determine the fidelity of subsequent regenerative progression. These regulatory inputs converge upstream of overt cell-cycle entry, underscoring early activation as a mechanistically distinct and vulnerable checkpoint within the regenerative trajectory.

### 2.4. The Aged MuSC Niche Constrains Early Activation and Introduces Delayed Activation Kinetics

A defining feature of muscle aging is a progressive decline in niche support for timely and coordinated MuSC activation. Rather than reflecting loss of MuSC number, age-associated regenerative failure increasingly arises from alterations in the local niche that constrain early activation competence [25,29,30]. Chronic inflammation [8,25], redox imbalance [30], and extracellular matrix remodeling [7,36,52,53] reshape the physical and biochemical environment encountered during early regeneration, increasing the stress load imposed at the activation checkpoint.

These niche-level alterations act primarily upstream of cell-cycle entry. Age-related changes in extracellular matrix composition and mechanical properties are sufficient to disrupt MuSC behavior, while restoration of youth-like matrix cues partially rescues regenerative performance in aged muscle [7,52]. In parallel, elevated tonic signaling within the aged niche destabilizes quiescence control and promotes inefficient or inappropriate activation, progressively eroding self-renewal capacity and delaying the onset of productive regeneration [37].

Early activation is also tightly coupled with metabolic readiness, which imposes an additional constraint on activation fidelity. Distinct metabolic states shape the ability of MuSCs to execute early activation programs and influence subsequent regenerative efficiency, providing a mechanism by which the aged niche biases activation toward delayed, asynchronous, or non-productive trajectories [50]. As a result, MuSC activation in aged muscle becomes slower and less synchronized across the population, reducing effective regenerative output even when MuSC numbers remain largely preserved [54].

These findings point to early activation as a rate-limiting checkpoint in aged muscle regeneration, where disrupted quiescence exit, altered niche sensing, and impaired metabolic preparedness converge to constrain regenerative competence (Figure 1B).

## 3. Early Activation as a Vulnerable Checkpoint in Aged Muscle

In aged murine muscle injury models, skeletal muscle regeneration declines with age despite largely preserved MuSC numbers, indicating functional impairment rather than MuSC depletion. Increasing evidence suggests that aged MuSCs exit quiescence less efficiently and with poorer coordination [6], entering a stress-burdened activation trajectory that precedes overt defects in proliferation or differentiation [8,36]. Under these conditions, early activation becomes a vulnerable checkpoint in which activation quality strongly influences downstream regenerative outcomes. Inefficient progression through this phase may transiently sustain regeneration via compensatory responses, while progressively eroding self-renewal capacity and long-term maintenance of the MuSC pool [5,12].

### 3.1. The Aged Niche Selectively Burdens Early Activation

Age-associated changes in the murine muscle microenvironment—including persistent oxidative stress, chronic inflammation, and progressive extracellular matrix stiffening—do not uniformly impair all stages of the myogenic program. Instead, growing evidence indicates that these alterations place a disproportionate burden on early activation [6], a phase that is particularly sensitive to niche-derived mechanical [7] and signaling cues required for coordinated exit from quiescence and reliable activation execution [37].

In aged murine muscle subjected to acute injury (e.g., cardiotoxin models), MuSCs more often exhibit delayed or unstable activation than an outright failure of proliferation [8]. Disruption at this stage is frequently associated with altered division patterns, reflecting impaired activation quality rather than intrinsic loss of proliferative capacity [34,35,36]. Consistent with this interpretation, ex vivo-engineered niche systems show that matrix stiffness and related mechanical cues directly regulate division mode and self-renewal behavior, underscoring the sensitivity of early activation to biophysical niche properties [55].

Signaling pathways that govern asymmetric division and long-term self-renewal, including p38α/β MAPK-dependent programs, act preferentially during the quiescence-to-activation transition [5]. This restricted temporal window makes early activation especially sensitive to niche perturbations, such that excess stress and distorted signaling in aged muscle disrupt the coordination and timing required for high-fidelity activation [5,12].

### 3.2. Transcriptomic Evidence for a Stage-Selective Defect in Aged MuSCs

To test whether aging imposes a stage-selective constraint consistent with an early activation checkpoint model, we re-examined the publicly available Gene Expression Omnibus (GEO) RNA-seq dataset GSE126665, which profiles fluorescence-activated cell sorting (FACS)-purified MuSCs isolated from uninjured young and aged mouse skeletal muscle [56]. Rather than focusing on individual differentially expressed genes, we evaluated predefined gene sets corresponding to discrete stages of the regenerative trajectory, including quiescence, early activation, proliferation, and differentiation. These stage-specific gene sets were defined a priori based on published stage markers and were applied uniformly across samples without re-weighting based on the current dataset (see Table 1 and Supplementary Table S1). Processed and normalized expression values as provided by the original study were used for comparative stage-level interpretation. Coordinated expression trends within each curated gene set were examined to assess relative shifts across regenerative states.

**Figure 2:**
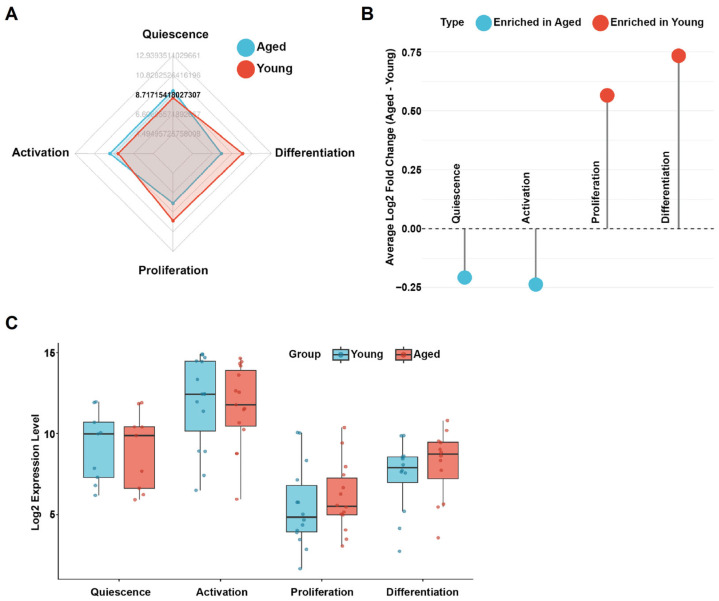
Stage-selective attenuation of early activation-associated transcriptional programs in aged muscle stem cells (MuSCs). (**A**) Radar plot showing relative gene set activity scores for quiescence, activation, proliferation, and differentiation programs in MuSCs isolated from young and aged mouse skeletal muscle (GSE126665). Compared with young cells, aged MuSCs show reduced activity in quiescence- and activation-associated transcriptional programs. (**B**) Mean log_2_ fold changes (aged vs. young) of stage-specific gene sets across the MuSC regenerative trajectory. Quiescence and activation programs are selectively attenuated in aged cells, whereas proliferation and differentiation programs show relative maintenance or enrichment. (**C**) Distribution of stage-specific gene set activity scores across individual samples. Aged MuSCs display lower activation-associated scores with increased variability, indicating less coordinated entry into early activation, while downstream transcriptional programs remain detectable in a subset of cells.

This analysis revealed a consistent and asymmetric pattern: transcriptional programs supporting quiescence maintenance and early activation were selectively attenuated in aged MuSCs, whereas gene sets associated with proliferation and differentiation were comparatively preserved (Figure 2A–C). This stage-selective transcriptional reweighting was reproducible across complementary analytical views, including aggregate stage-level activity scores, enrichment distributions, and per-sample comparisons, arguing against a global suppression of myogenic capacity with age.

Instead, these data indicate that age-associated regenerative decline is linked primarily to reduced transcriptional support for entry into the regenerative program rather than loss of downstream myogenic competence. Proliferation- and differentiation-associated programs remain accessible in the subset of aged MuSCs that successfully traverse early activation, consistent with early activation functioning as a selective and failure-prone checkpoint rather than a uniform collapse of myogenic potential [56].

### 3.3. Compensatory Proliferation May Mask Early Failure While Potentially Accelerating Long-Term Attrition

A stage-selective defect in early activation provides a mechanistic explanation for a recurring paradox in aged muscle regeneration: markers of proliferation and differentiation remain detectable after injury, yet regenerative capacity progressively declines with repeated repair cycles. When early activation is inefficient, regenerative demand is met disproportionately by the subset of MuSCs that successfully traverse this checkpoint, rather than by uniform recruitment of the MuSC pool. This compensatory reliance can sustain short-term regeneration but does so at the cost of long-term MuSC maintenance.

Durable self-renewal depends on tightly regulated signaling during the quiescence-to-activation transition, a window that is particularly vulnerable in aged MuSCs. Consistent with this framework, age-associated dysregulation of p38α/β MAPK signaling impairs asymmetric division and reduces the generation of quiescent daughter cells, thereby accelerating MuSC attrition across repeated rounds of regeneration [5,8,12].

These observations indicate that compensatory proliferation may mask early activation failure while simultaneously driving progressive depletion of the MuSC pool. Regenerative decline in aged muscle therefore reflects defects arising upstream of proliferation and differentiation, rather than a primary loss of downstream myogenic capacity.

## 4. MG53: Beyond Membrane Repair

The preceding sections identify early satellite cell activation as a stress-sensitive transitional checkpoint that may represent a critical constraint in aged muscle regeneration. This perspective redirects focus from augmenting downstream proliferative output to-ward maintaining activation fidelity during the quiescence-to-activation transition under stress. Based on its established roles in membrane repair and stress signaling in striated muscle and other tissues, MG53 emerges as a candidate whose stress-responsive, membrane-associated functions may be relevant to the physiological demands of early activation in aging muscle.

### 4.1. Canonical Roles of MG53 in Skeletal Muscle

MG53 is a striated muscle–enriched tripartite motif protein best known for its essential role in sarcolemmal membrane repair [14]. In skeletal myofibers, MG53 rapidly senses membrane disruption and nucleates repair complexes at injury sites, coordinating vesicle recruitment and membrane resealing under contractile stress [13,49].

MG53 contains an N-terminal RING domain, followed by a B-box, coiled-coil region, and a C-terminal PRY/SPRY domain that supports protein–protein interactions and higher-order assembly. Recent structural studies demonstrate that coordinated domain interactions enable MG53 oligomerization and membrane-associated activation, both of which are required for efficient membrane repair [57,58].

Beyond its scaffolding role, MG53 also functions as a RING-dependent E3 ubiquitin ligase. A well-characterized substrate is insulin receptor substrate-1 (IRS-1), whose ubiquitination by MG53 dampens IGF/insulin signaling and modulates myogenic progression in a context- and timing-dependent manner [59,60]. These signaling functions position MG53 not only as a structural repair protein but also as a membrane-proximal regulator of stress-responsive intracellular pathways.

Although age-dependent transcriptional changes in MG53 remain incompletely defined, several studies report impaired membrane repair efficiency and altered MG53 subcellular compartmentalization in aged muscle, suggesting functional decline rather than uniform reduction in MG53 transcript abundance [16,61]. These observations are consistent with the possibility that MG53 activity may be compromised through changes in localization, post-translational regulation, or stress environment in aging muscle.

MG53 is likewise expressed in cardiac muscle, where it contributes to membrane repair in cardiomyocytes [61]. Evidence from metabolic and inflammatory models further implicates MG53 in the modulation of insulin signaling and in the regulation of immune and vascular stress responses [22,62]. These extra-muscular functions further support its role as a stress-responsive and membrane-proximal signaling regulator.

These properties define MG53 as a membrane-proximal, stress-responsive regulator that couples membrane perturbation to adaptive control of intracellular signaling. This dual capacity provides a mechanistic rationale for considering MG53 function beyond terminally differentiated myofibers, particularly during early MuSC activation—a phase marked by rapid membrane remodeling and heightened sensitivity to stress and signaling imbalance.

### 4.2. Activation-Associated Stress, Membrane Remodeling, and Signaling Imbalance in Aged MuSCs

Early MuSC activation precedes cell-cycle entry and requires tight temporal coordination of membrane remodeling, cytoskeletal reorganization, and integration of growth factor and stress signaling. This transition is intrinsically stress-sensitive, as rapid membrane deformation and structural reorganization transiently increase susceptibility to ionic imbalance, Ca^2+^ influx, and oxidative perturbations [10,63]. In young muscle, these transient stresses are efficiently buffered, allowing coordinated execution of early activation programs [10,49]. In contrast, aging exacerbates these constraints through sustained oxidative stress, chronic inflammatory signaling, and altered extracellular matrix mechanics, thereby reducing tolerance to activation-associated perturbations [5,7,8].

**Figure 3:**
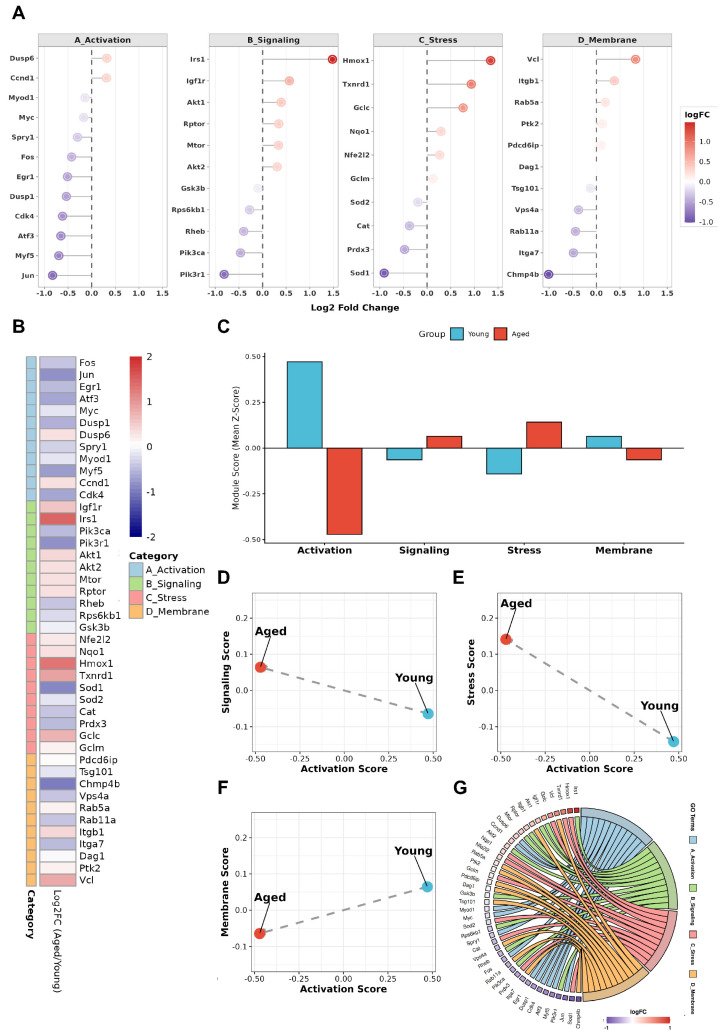
Functional shifts in early activation-associated programs in aged muscle stem cells (MuSCs). Module-level analysis reveals selective attenuation of activation-associated programs in aged MuSCs, accompanied by relative enrichment of stress and signaling modules. (**A**) Dot plots showing log_2_ fold changes (aged vs. young) of representative genes grouped into four functionally modules relevant to early MuSC activation: immediate-early activation genes (A_Activation), growth factor and metabolic signaling pathways (B_Signaling), intrinsic stress-response programs (C_Stress), and membrane-associated remodeling and adhesion-related processes (D_Membrane). Dot size indicates statistical significance; color reflects direction and magnitude of expression change. (**B**) Heatmap showing gene-level expression changes within each functional module. Genes were selected based on established functional annotation and prior literature to represent core module features. (**C**) Module activity scores summarizing the mean Z-scored expression of genes within each module in young and aged MuSCs. (**D**–**F**) Pairwise comparisons of module activity scores reveal inverse relationships between activation-associated modules and stress- or signaling-associated programs, highlighting divergent shifts in early activation state between young and aged MuSCs. (**G**) Chord diagram illustrating cross-module gene–function relationships, emphasizing coordinated module-level changes rather than isolated gene-specific effects.

To assess whether aging alters the balance of processes supporting early activation, we reanalyzed the GSE126665 dataset using predefined, functionally anchored gene modules rather than individual differentially expressed genes [56]. Modules were defined prior to MG53-focused interpretation and applied uniformly across samples. These modules represent activation-relevant processes, including immediate early activation responses [10,42,44,64], PI3K–AKT–mTOR signaling [9,59,65], NRF2-mediated oxidative buffering [7,49,66,67], and membrane remodeling programs [15,68,69,70] (see Supplementary Table S2 for full annotation). Aged MuSCs exhibited a coordinated shift in module usage (Figure 3A–G), characterized by reduced activation- and membrane-remodeling programs and relative enrichment of stress-response-associated modules, including those annotated for membrane repair and stress regulation.

Across samples, activation- and membrane-remodeling scores were inversely related to stress- and membrane-repair-associated signaling scores. This pattern is consistent with aged MuSCs initiating early activation under transcriptional conditions characterized by elevated stress and altered signaling balance rather than coordinated activation-supportive remodeling. Under such conditions, diminished membrane-remodeling programs may sensitize cells to Ca^2+^ influx and secondary oxidative cascades during activation [71,72]. Thus, activation failure in aged MuSCs may reflect a stress-biased imbalance that disrupts the coordination and timing of early activation, rather than a generalized loss of myogenic capacity. To date, direct measurements of MG53 dynamics during stage resolved MuSC activation are limited. The following section therefore integrates established MG53 functions in muscle and stress biology with the physiological demands of early activation to generate a testable framework, rather than to assert stage-specific causality.

### 4.3. MG53 as a Permissive Regulator at the Early Activation Checkpoint

The reciprocal patterns observed in Figure 3 indicate that early activation failure in aged muscle reflects instability of the cellular conditions required for coordinated activation, rather than loss of intrinsic myogenic fate programs. We therefore propose a model in which MG53 functions as a permissive regulator at the early activation checkpoint. Rather than directing lineage commitment, MG53 may constrain damage amplification and signaling imbalance during the quiescence-to-activation transition, thereby supporting coordinated execution of early activation under stress. This framework integrates established MG53 stress-regulatory functions with the physiological constraints of early activation and remains to be directly tested in stage-resolved MuSC systems.

#### 4.3.1. MG53-Mediated Buffering of Oxidative and Mitochondrial Stress During Early Activation

Early MuSC activation imposes acute oxidative and metabolic demands driven by rapid membrane remodeling, transient Ca^2+^ flux, and increased mitochondrial activity prior to cell-cycle entry. These processes render MuSCs particularly vulnerable to secondary oxidative stress during the activation interval. Consistent with this vulnerability, multiple studies have shown that MG53 preserves cellular integrity under oxidative challenge by limiting damage propagation and supporting mitochondrial and membrane-associated homeostasis [21,66,73]. In cardiomyocytes and skeletal muscle, MG53 attenuates reactive oxygen species-induced injury, preserves mitochondrial membrane potential, and suppresses downstream cell death pathways under ischemic or oxidative stress conditions [66,74]. Such stress-buffering capacity may increase tolerance to activation-associated oxidative perturbations, stabilizing early activation without directly instructing downstream fate specification. Although direct studies measuring MG53-dependent changes in early activation kinetics in satellite cells remain limited, the consistent demonstration of MG53-mediated mitochondrial and redox stabilization under oxidative stress provides mechanistic plausibility for its role during the activation interval.

#### 4.3.2. MG53 Modulation of Inflammatory Signaling and Stress Amplification

The aged muscle niche is characterized by chronic low-grade inflammation that intersects with early MuSC activation to amplify stress signaling and compromise activation fidelity. Consistent with a role in stress containment, emerging evidence indicates that MG53 can attenuate inflammatory signal propagation by modulating innate immune-associated pathways [61]. Mechanistic studies further show that MG53 limits excessive interferon-β production and inflammatory cytokine release by regulating ryanodine receptor-dependent Ca^2+^ handling and downstream stress signaling cascades, thereby constraining secondary amplification of inflammatory stress [61,75].

These observations are consistent with MG53 acting upstream of transcriptional fate programs, acting instead on the signaling environment in which early activation occurs. In vivo administration of recombinant MG53 has been shown to reduce inflammation-associated tissue damage across diverse stress contexts, including viral infection, ischemia–reperfusion injury, and sterile tissue stress [76,77]. Although direct evidence for MG53-mediated inflammatory regulation in MuSCs remains limited, these findings support a model in which MG53 contributes to limiting inflammatory noise during early activation, thereby preventing stress-dominated signaling states that destabilize the timing and coordination of activation.

#### 4.3.3. MG53-Dependent Membrane Stabilization at the Activation Checkpoint

Membrane integrity constitutes a critical physical constraint during early MuSC activation, when cells undergo rapid shape change, adhesion remodeling, and cytoskeletal reorganization. Even modest membrane perturbations at this stage can amplify Ca^2+^ influx and oxidative signaling, biasing activation toward stress-dominated trajectories. Seminal studies identified MG53 as a nucleator of membrane repair complexes following mechanical or oxidative membrane disruption [13,14], and subsequent work demonstrated that exogenous MG53 enhances membrane resilience and limits damage propagation across ischemic, mechanical, and inflammatory injury contexts [16,78].

At the activation checkpoint, insufficient membrane stabilization may reinforce Ca^2+^-dependent and oxidative stress signaling, further destabilizing activation execution by analogy to established roles in differentiated myofibers. By constraining membrane-associated damage propagation, MG53 is well positioned to stabilize the physical execution of early activation. Together, these observations support the conceptualization of MG53 as a context-dependent, permissive regulator operating at the early activation checkpoint to support activation fidelity under stress. While membrane repair has not been directly examined in the context of activation timing, the physical demands of early activation make membrane stability a critical permissive factor.

### 4.4. Early Activation Fidelity as a Determinant of Regenerative Aging

**Figure 4:**
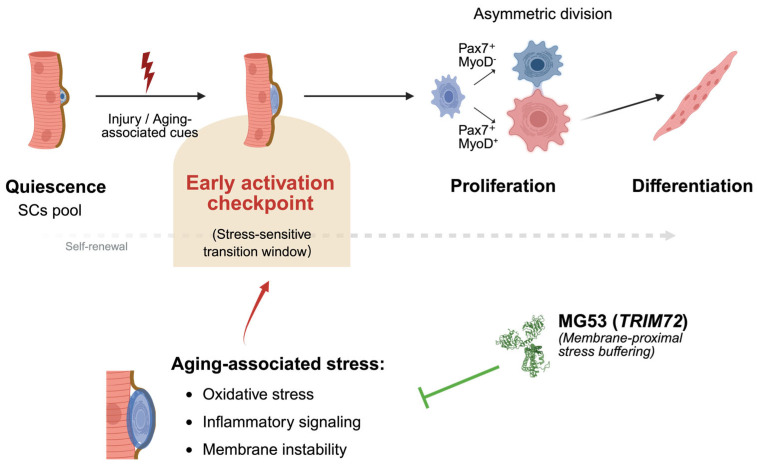
Conceptual model of MG53 as a permissive stabilizer of early activation fidelity in aged muscle. Schematic illustrating a stage-selective model of muscle stem cell (MuSC) dysfunction during aging and the proposed role of MG53 (*TRIM72*) at the early activation checkpoint. Under permissive conditions, MuSCs transition from quiescence through a stress-sensitive early activation window, enabling coordinated asymmetric division, self-renewal, and productive differentiation. With aging, persistent oxidative stress, chronic inflammatory signaling, and intrinsic membrane instability increase the stress burden encountered during early activation. These factors destabilize the coordination and timing of the activation checkpoint, reducing activation fidelity and biasing MuSCs toward delayed or inefficient activation trajectories, despite relative preservation of downstream proliferative and differentiation capacity. Within this stress-limited context, thereby improving coordination under stress rather than instructing lineage commitment, MG53 may increase the likelihood of successful passage through the early activation checkpoint and supporting long-term self-renewal and regenerative capacity in aged muscle. Created with BioRender.com (2026 version); https://BioRender.com/g74wago (accessed on 11 January 2026).

Age-associated decline in skeletal muscle regeneration is best explained not by a uniform loss of myogenic programs, but by a progressive reduction in fidelity at the early activation checkpoint [2,36] (Figure 4). Although proliferative and differentiation programs often remain accessible, instability during the quiescence-to-activation transition disrupts the coordination and timing required for sustained regenerative output [8].

Early activation imposes substantial organizational demands on MuSCs, requiring tightly coordinated membrane remodeling, cytoskeletal reorganization, metabolic priming, and signal integration within a rapidly changing niche [10,11]. Aging selectively intensifies oxidative, inflammatory, and mechanical stress during this phase, when tolerance is inherently limited [37]. Consequently, MuSCs are more likely to enter delayed or poorly synchronized activation trajectories that impair asymmetric division and long-term self-renewal, even when downstream myogenic programs remain intact [5,8,34].

Within this view, MG53 and related factors do not act as lineage-specifying regulators, but as permissive stabilizers that limit damage amplification and signaling noise during early activation [14,31,72]. By supporting the fidelity of the quiescence-to-activation transition rather than directing fate choice, these mechanisms increase the likelihood that MuSCs successfully traverse the activation checkpoint under stress. This perspective helps explain why interventions focused solely on enhancing proliferation or differentiation often yield limited benefit in aged muscle [5,8,36]. Increased proliferative output in aging frequently reflects compensatory activation of a restricted subset of MuSCs and is accompanied by impaired asymmetric division and accelerated MuSC pool attrition, rather than durable restoration of regenerative capacity [5,8]. When instability originates upstream at the activation checkpoint, downstream programs unfold within a distorted temporal and signaling context, constraining the efficacy of late-stage interventions [7]. In contrast, preserving early activation fidelity targets the key candidate bottleneck through which regenerative potential must pass [36].

By centering regenerative aging on the quality of early activation, Figure 4 integrates niche-derived stress, intrinsic execution capacity, and permissive stabilizers into a unified framework in which coordination under stress—rather than maximal output—emerges as a potential constraint on MuSCs-mediated regeneration.

## 5. Implications for Aged Muscle Regeneration and Therapeutic Perspectives

Reframing age-associated regenerative decline as a failure of stress-limited early activation, rather than a uniform loss of myogenic capacity, has important therapeutic implications for aged skeletal muscle [4,12,36]. If instability at the quiescence-to-activation transition represents a candidate bottleneck, strategies focused primarily on enhancing downstream proliferation or differentiation may be insufficient to restore durable regeneration and could potentially accelerate MuSC exhaustion by repeatedly engaging a restricted subset of MuSCs capable of traversing early activation [5,7,8].

It should be noted that the conceptual framework discussed here is derived predominantly from studies in murine injury and aging models. Direct evidence for a stage-selective activation checkpoint or MG53-dependent stabilization in human satellite cells remains limited. Extrapolation to human aging muscle must be made cautiously. Future validation in primary human MuSCs and aged human skeletal muscle tissue will be essential to assess translational relevance.

This perspective shifts therapeutic focus toward stabilizing the activation window itself—a brief but stress-sensitive phase preceding cell-cycle entry [10]. In this context, MG53 can be considered a candidate stress-buffering factor whose established roles in membrane repair and redox-sensitive damage control may constrain amplification of oxidative, inflammatory, and mechanically induced stress during early activation [15,66,71,78]. The predicted benefit of MG53 lies not in sustained enhancement of myogenic signaling, but in transiently improving activation fidelity.

Although recombinant MG53 provides tissue protection across multiple injury models [13,61], therapeutic application in aged muscle would likely require precise temporal control and muscle-restricted exposure to minimize potential interference with insulin/IGF signaling and systemic metabolic regulation [17,59,60]. Circulating MG53 may reflect global membrane stress burden [28], but is unlikely to serve as a direct indicator of MuSC activation competence [13].

Together, these considerations underscore the importance of clearly defining the experimental contexts in which the proposed model has been validated before advancing translational strategies.

## 6. Open Questions and Future Directions

Despite increasing interest in early MuSC activation as a vulnerable stage in aged muscle regeneration, several important questions remain.

First, it is still unclear where and how MG53 acts during early activation. MG53 may function within MuSCs, act indirectly through myofibers or niche components, or influence activation through interactions between these compartments. Distinguishing cell-autonomous from niche-mediated effects will require temporally controlled and cell type-specific perturbation models.

Second, early activation remains poorly defined in practical terms. This phase is often inferred from later markers of proliferation or differentiation, yet it likely includes a specific sequence of membrane remodeling, cytoskeletal changes, and stress signaling that occurs before cell-cycle entry. Approaches that resolve these events over time and across modalities will be needed to distinguish true activation from quiescence exit or early proliferation, and to identify features that predict successful regeneration.

Third, it remains uncertain whether improving early activation is sufficient to enhance regeneration without compromising long-term MuSC maintenance. How changes in activation dynamics affect asymmetric division, self-renewal, and cumulative proliferative history have not been fully addressed. Answering these questions will be essential for evaluating whether stabilizing early activation can provide durable benefits in aged muscle.

From a translational perspective, extending these findings to human systems represents a critical next step. Human MuSCs exhibit age-associated alterations in metabolic state, inflammatory exposure, and niche mechanics that may not fully recapitulate murine models. Integration of single-cell transcriptomics, ex vivo analyses of aged human muscle biopsies, organoid systems, and functional assays assessing membrane repair capacity, redox resilience, and activation kinetics will be essential to determine whether activation checkpoint instability constitutes a conserved and therapeutically actionable feature of human muscle aging.

## 7. Conclusions

Early MuSC activation represents a critical and stress-sensitive step in skeletal muscle regeneration that is selectively destabilized with aging. Rather than reflecting a global loss of myogenic potential, age-associated regenerative decline is better explained by reduced fidelity during the quiescence-to-activation transition, when cells must coordinate membrane remodeling, stress adaptation, and signaling integration under increased burden. Within this view, MG53 is most plausibly positioned not as a fate-determining factor, but as a permissive regulator that supports the execution of early activation by limiting damage amplification and preserving coordination under stress. Although key mechanistic questions remain, this perspective underscores activation fidelity—rather than downstream proliferative output—as a central determinant of regenerative capacity in aging skeletal muscle.

## Data Availability

This review includes secondary analyses of publicly available datasets cited in the manuscript. The code used for data processing and analysis is publicly available at https://github.com/jethro-wang-zs/MG53_on_MuSCs (accessed on 2 December 2025). No new primary data were generated in this review.

## Supplementary Materials

The following supporting information can be downloaded at: https://www.mdpi.com/article/10.3390/cells15050463/s1, Table S1: Literature-curated gene sets defining stage-specific MuSC states during skeletal muscle regeneration; Table S2: Functional annotation and literature support for curated gene modules used in transcriptomic reanalysis.
