# Supplementary material for: MG53 in Early Skeletal Muscle Stem Cell Activation: Implications for Aged Muscle Regeneration
Source: Cells. 2026 Mar 5;15(5):463. doi: 10.3390/cells15050463 (PMC12984147; doi:10.3390/cells15050463)
Supplement: Supplementary file 1 [file cells-15-00463-s001.zip › cells-4141900-supplementary.pdf]

Supplementary

MG53 in Early Skeletal Muscle Stem Cell Activation: Implications for Aged Muscle Regeneration

Supplementary Table S1. Literature-curated gene sets defining stage-specific MuSC states during skeletal muscle regeneration.

Gene sets were defined a priori based on published lineage-tracing, in vivo activation kinetics, and transcriptomic studies of skeletal muscle regeneration. Genes were selected to represent canonical markers of quiescence, early activation, proliferation, and differentiation. These curated gene sets were not derived from de novo clustering of the analyzed dataset, thereby minimizing circular interpretation of differential expression patterns. Literature support for each gene is provided in the reference column.

Gene	Biological role	Literature reference
**1. Quiescence gene set**		
Pax7	Canonical satellite cell identity marker	
Notch3	Notch signaling in quiescence maintenance	
Rbpj	Notch downstream effector	
Spry1	Maintains return to quiescence	
Foxo3	Stress resistance & quiescence preservation	[38–41]
Vcam1	Adhesion molecule in quiescent SCs	
Calcr	Recently described quiescent SC marker	
Sdc4	Niche adhesion receptor	
Col5a3	ECM-associated quiescent niche component	
**2. Early Activation gene set (immediate-early + stress-adaptive + early commitment genes)**		
Fos	Immediate early gene	
Jun / Junb	Immediate early	
Egr1	Early activation transcription factor	
Atf3	Stress-induced activation regulator	
Myf5	Early myogenic commitment	[10,11,32,42–46]
Myod1	Commitment regulator	
Mt1 / Mt2	Metal-responsive stress genes	
Hmox1	Oxidative stress response	
Socs3	Cytokine feedback regulator	

Cxcl1/2/5	Injury response chemokines	
Hmgb2	Chromatin remodeling during activation	
**3. Proliferation gene set**		
Mki67	Proliferation marker	
Pcna	DNA replication	
Ccnd1	G1 progression	
Cdk4	G1 kinase	
Ccnb1	G2/M transition	
Cdc20	APC/C regulator	
Aurka / Aurkb	Mitotic kinases	[9,10,44,47,48]
Plk1	Mitotic regulator	
Cdc6	DNA licensing	
Cdk2	S phase kinase	
Top2a	DNA topology	
Birc5	Survivin	
**4. Differentiation gene set**		
Myog	Terminal differentiation TF	
Myf6	Myogenic differentiation factor	
Myh3	Embryonic MyHC	
Myh8	Perinatal MyHC	
Des	Desmin, structural	
Mef2c	Myogenic TF	[32,51]
Tnni2	Contractile protein	
Mylpf	Myosin light chain	
Myh1	Fast-type IIx myosin isoforms	
Myh2	Fast-type IIa myosin isoforms	
Tnnt3	Troponin T isoform in fast-twitch fibers	
Acta1	Principal actin isoform in adult skeletal muscle	

Supplementary Table S2. Functional annotation and literature support for curated gene modules used in transcriptomic reanalysis

Gene	Functional role in MuSC biology	Literature support
**A_Activation module**		
Fos	Immediate-early transcription factor induced upon injury	
Jun	Immediate-early response gene	
Egr1	Early activation transcription factor	
Atf3	Stress-responsive regulator of activation	
Dusp1	MAPK feedback regulator during stress signaling	
Dusp6	ERK pathway modulation	[10,42,44,64]
Myf5	Early myogenic commitment factor	
Myod1	Myogenic determination regulator	
Myc	Metabolic priming during activation	
Ccnd1	Early G1 cell-cycle priming	
Cdk4	G1 kinase activation	
Spry1	Regulator of quiescence–activation balance	
**B_Signaling module (PI3K–AKT–mTOR axis)**		
Irs1	IGF/insulin signaling adaptor	
Igf1r	Growth factor receptor	
Pik3ca	PI3K catalytic subunit	
Pik3r1	PI3K regulatory subunit	
Akt1	AKT signaling mediator	
Akt2	AKT isoform	[9,59,65]
Mtor	mTOR kinase	
Rptor	mTORC1 component	
Rps6kb1	mTORC1 downstream effector	
Rheb	mTOR activator	
Gsk3b	Growth signaling integrator	
**C_Stress module (NRF2–oxidative stress axis)**		
Nfe2l2	Master regulator of antioxidant response (NRF2)	
Hmox1	Oxidative stress response enzyme	
Gclc	Glutathione synthesis	
Gclm	Glutathione synthesis	
Sod1	ROS detoxification	
Sod2	Mitochondrial ROS detoxification	[7,49,66,67]
Cat	Catalase	
Prdx3	Mitochondrial antioxidant	
Txnrd1	Thioredoxin pathway	
Nqo1	Redox buffering enzyme	
**D_Membrane module (Integrin–ESCRT–membrane remodeling axis)**		
Itga7	Integrin-mediated niche adhesion	[15,68–70]

Itgb1	Integrin signaling
Dag1	Dystroglycan complex (ECM anchoring)
Vcl	Focal adhesion scaffold
Ptk2 (FAK)	Integrin downstream signaling
Rab5a	Endosomal trafficking
Rab11a	Recycling endosome
Tsg101	ESCRT-I complex
Vps4a	ESCRT ATPase
Chmp4b	ESCRT-III component
Pdcd6ip (ALIX)	ESCRT adaptor
